# Supplementary material for: Eukaryotic-driven directed evolution of Cas9 nucleases
Source: Genome Biol. 2024 Mar 25;25:79. doi: 10.1186/s13059-024-03215-9 (PMC10962177; doi:10.1186/s13059-024-03215-9)
Supplement: Supplementary file 1 — Additional file 1: Fig. S1. Yeast cultures from the auxotrophic evolution platform. Fig. S2. TetR-EGFP reporter screening platform. Fig. S3. Editing activity of selected variants in endogenous loci of HEK293 cells. Fig. S4. 3D position of mutations in UltraCjCas9. Fig. S5. In vitro PAM characterization of CjCas9 WT and UltraCjCas9. Fig. S6. List of PAM sequences and editing at GUIDE-Seq ON-target sites. Fig. S7. Editing activity of CjCas9 WT and UltraCjCas9 in the PDCD1 locus using mismatched sgRNAs. [file 13059_2024_3215_MOESM1_ESM.pdf]

Additional file 1: Supplementary Information

Eukaryotic-driven directed evolution of Cas9 nucleases

Giulia Vittoria Ruta[1], Matteo Ciciani[1,2], Eyemen Kheir[1], Michele Domenico Gentile[1], Simone Amistadi[1#], Antonio Casini[3], Anna Cereseto[1]

Affiliations:

[1] Department CIBIO, Laboratory of Molecular Virology, University of Trento, Italy

[2] Department CIBIO, Laboratory of Computational Metagenomics, University of Trento, Italy

[3] Alia Therapeutics s.r.l, Trento, Italy

[#] Current address: Université de Paris, Imagine Institute, Laboratory of chromatin and gene regulation during development, INSERM UMR 1163, Paris, France

Figure S1

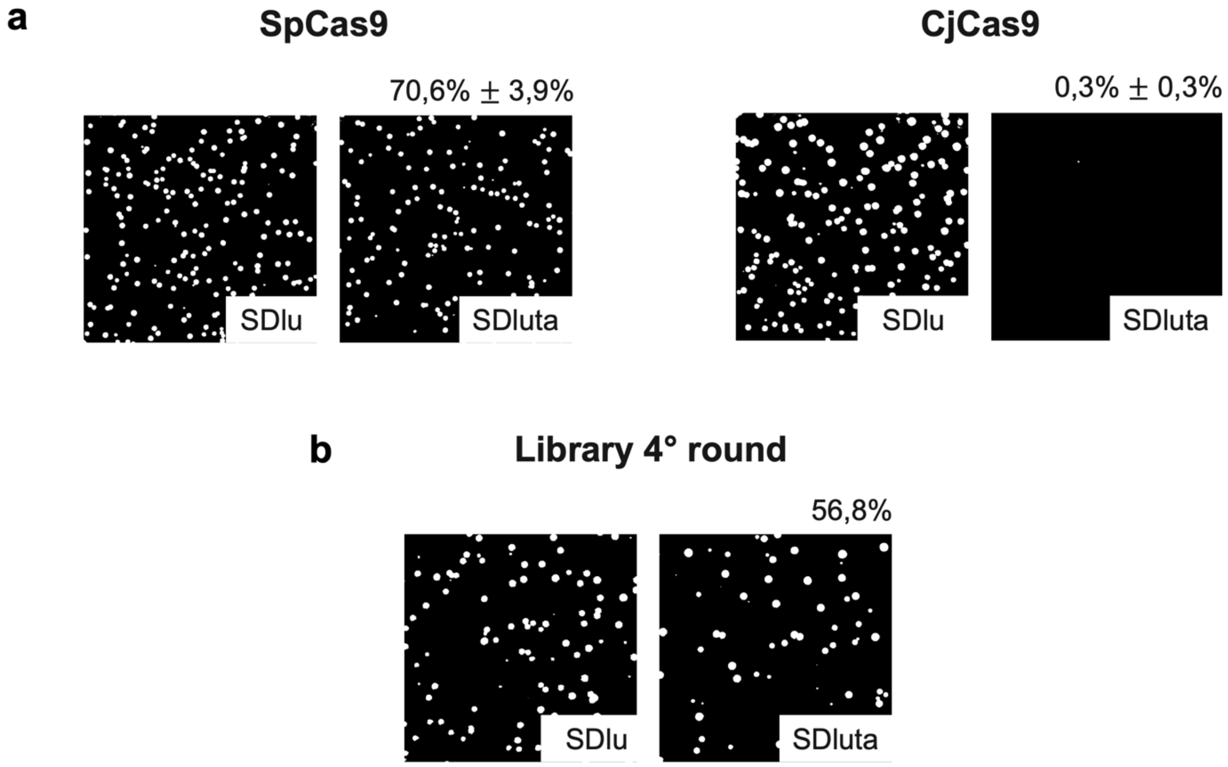

Figure S1. Yeast cultures from the auxotrophic evolution platform. (**a**) Representative plates obtained by transforming yeasts with SpCas9 and CjCas9 plasmids. Reported percentages are means ± standard deviation of colony survival for n=3 biologically independent replicates. Survival rates are determined as ratios of colonies growing in SDluta (cleaved cassettes) and SDlu (transformants). (**b**) Representative plates showing the survival rate of yeast colonies after transformation with the 4th round library as a PCR amplicon. The mean percentage of survival is reported above the panels.

Figure S2

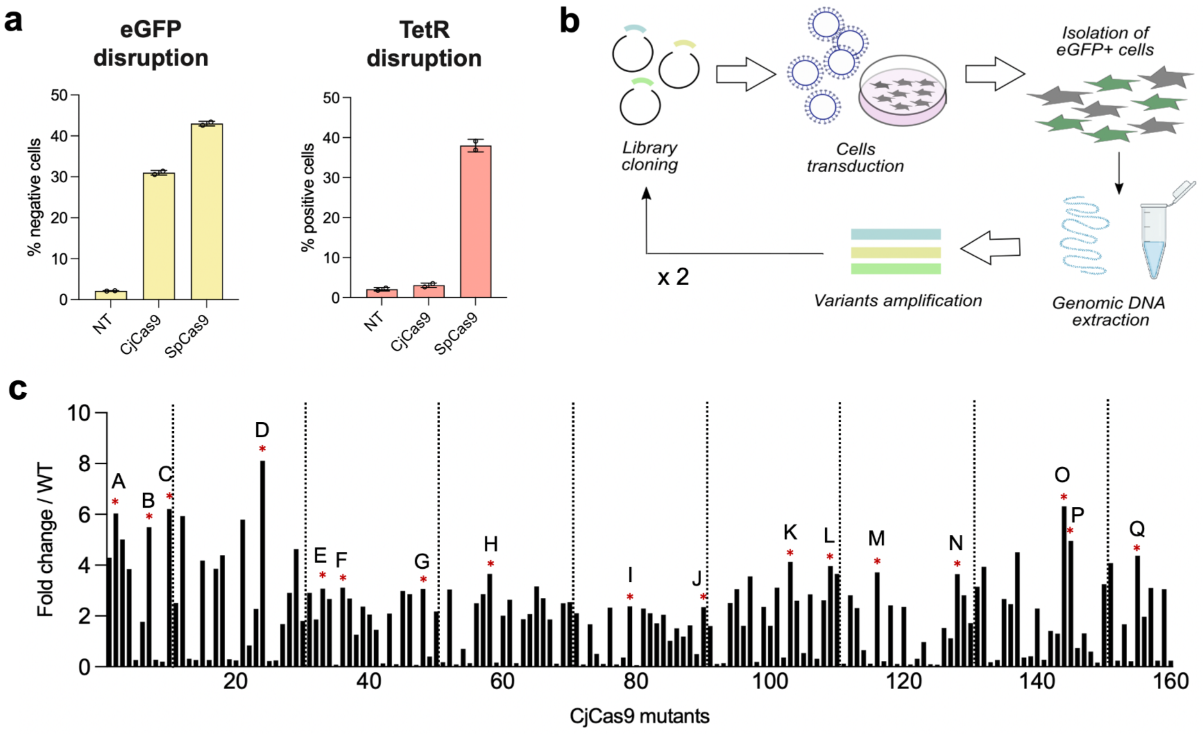

Figure S2. TetR-EGFP reporter screening platform. (**a**) Validation of the TetR-EGFP reporter cell line with CjCas9 and SpCas9, compared with EGFP disruption assay. The TetR disruption assay exhibits a wider dynamic range compared to the eGFP disruption assay. Data shown as percentages of EGFP negative or positive cells obtained by FACS analysis 6 days after transfection with CjCas9 and SpCas9 targeting EGFP or TetR. Data are reported as mean ± standard deviation of n=2 biologically independent samples. Individual values are represented as empty circles. (**b**) Schematic representation of the screening rounds in the TetR-EGFP reporter cell line. Variants are amplified at each round and cloned in lentiviral vectors for transduction of the reporter cell line and isolation of EGFP positive cells by sorting. The process was repeated twice. (**c**) Test of cleavage activity of n=160 randomly picked CjCas9 mutants from the 4th round library after enrichment screening in mammalian cells using the TetR reporter system. Data are reported as fold changes of the

percentages of EGFP positive cells with respect to CjCas9 WT; values were obtained by FACS analysis 6 days after transfection of the reporter cell line with each variant plasmid. Dotted lines distinguish different groups of transfections. Considering the high variability between different groups, the best performing variants of each group (pointed out by red asterisks and letters) were selected for further characterization.

Figure S3

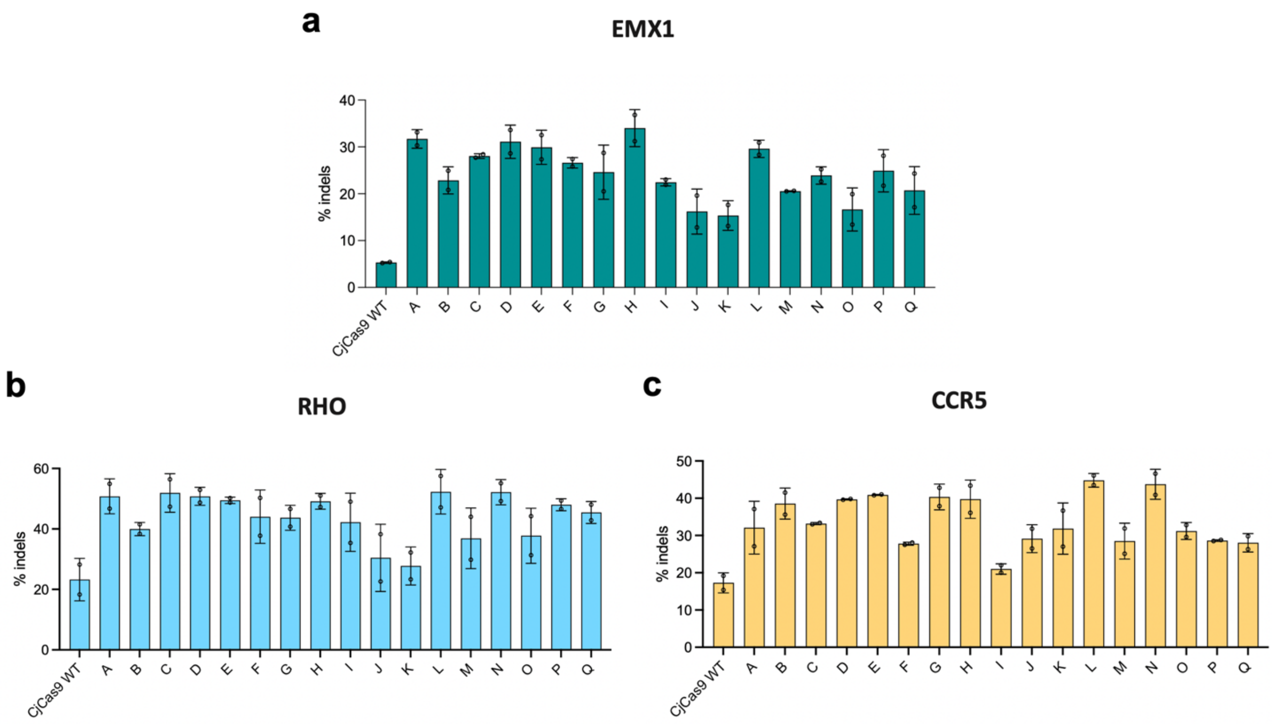

Figure S3. Editing activity of selected variants in endogenous loci of HEK293 cells. (**a**) DNA cleavage of the *EMX1* endogenous locus with CjCas9 and selected variants (mutants A to Q) after transient transfections. (**b**) DNA cleavage of *RHO* endogenous locus with CjCas9 and selected variants (mutants A to Q). (**c**) DNA cleavage of *CCR5* endogenous locus with CjCas9 and selected variants (mutants A to Q). Data reported as mean ± standard deviation of indels percentages of n=2 biologically independent samples. Individual values are represented as empty circles.

Figure S4

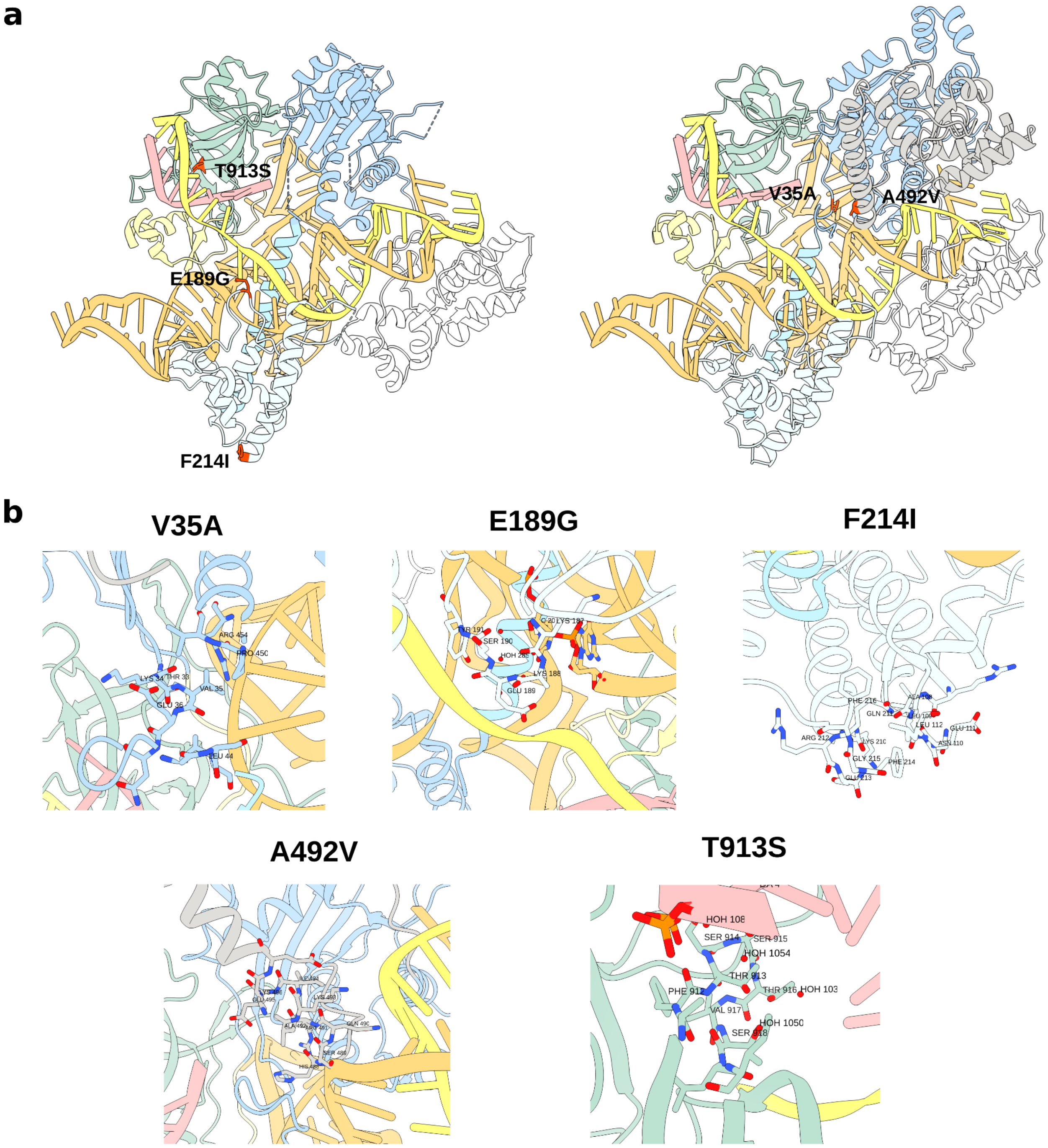

Figure S4. 3D position of mutations in UltraCjCas9. (**a**) Representation of key residues mutated in UltraCjCas9 (mutant H). Amino acids 189, 214 and 913 were highlighted in red in the crystal structure of CjCas9 (PDB ID 5X2H) using UCSF ChimeraX. Residues 35 and

492, which are missing from the crystal structure, are highlighted in red in the predicted structure of CjCas9 retrieved from the AlphaFold Protein Structure Database (accession Q0P897). CjCas9 domains are reported using the color scheme of the protein structure shown in **Figure 3a**. **b)** Zoomed-in view of each mutated residue and surrounding area.

Figure S5

a

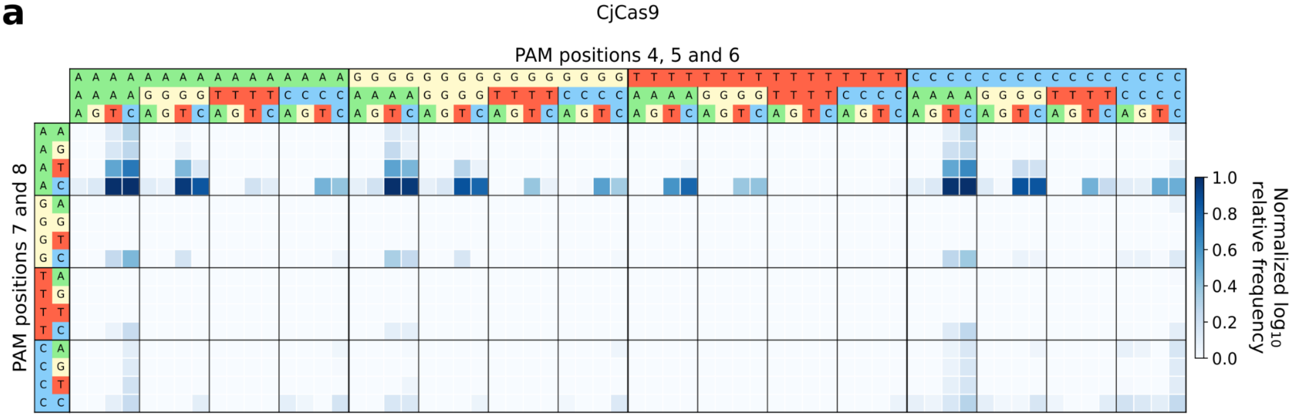

b

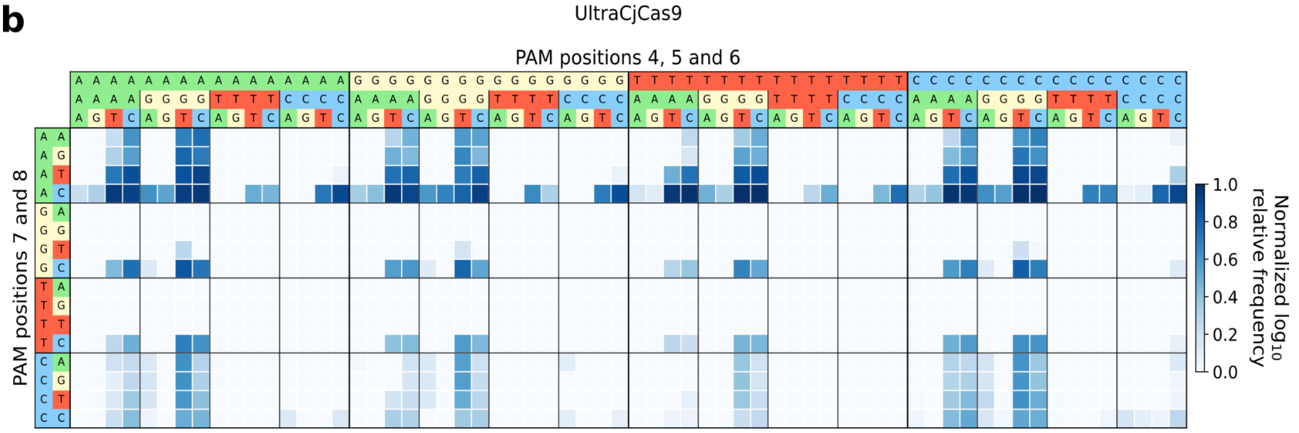

Figure S5. ***In vitro* PAM characterization of CjCas9 WT and UltraCjCas9.** Heatmaps representation of the PAM preference of CjCas9 WT (**a**) and UltraCjCas9 (**b**) obtained using an *in vitro* PAM determination assay. The PAM of CjCas9 can be summarized as a consensus sequence N_3VRYAC, as previously reported [15]. UltraCjCas9 exhibits a relaxed PAM, corresponding to the consensus sequence N_4RYAY.

Figure S6

a

VRYAC	
HEK site 2	AATAC
HBB	
BCR	CATAC
FAS1	CGTAC
HBG1	GATAC
CCR5	GGTAC
TRAC	
RHO	AGTAC
HPRT1	
EMX1	AGCAC
FANCF	
CHR8	AACAC
ZSCAN2	CGCAC
HEK site 1	GGCAC
TRBC1	
IL2RG	GACAC
VEGFA2	
PDCD1	CACAC
VEGFA3	

Not VRYAC	
CHR6	AACAT
ATM	AACAA
HEK site 3	AACAG
HEK site 4	TACAC

b

**TRBC**	CAGCGCACACCATGAAGGAGAATTGGGCAC
**TRAC**	TCACTGGATTTAGAGTCTCTCAGCTGGTAC
**HEK site 1**	GGGCAAGTGCTGTCTGCCATGCGCCGGCAC
**HEK site 2**	AACTTCCCAAGTGAGAAGCCAGTGGAATAC
**FANCF**	CCGAATTCCCCAGAAGCCAGTGGACTAGCAC

c

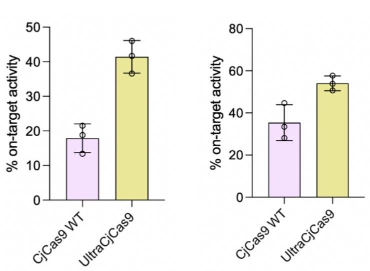

Figure S6. List of PAM sequences and editing at GUIDE-Seq ON-target sites. (**a**) List of PAM sequences selected to design sgRNAs targeting genomic loci. N_3VRYAC and not N_3VRYAC PAM sequences were chosen to widely evaluate the editing efficiency of UltraCjCas9. sgRNAs are listed in **Additional file 2: Table S4**. (**b**) DNA sequences representing the relative positions of SpCas9 and UltraCjCas9 sgRNAs targeting 5 selected genomic loci. PAM sequences of SpCas9 are underlined in gray, PAM sequences of UltraCjCas9 in yellow. (**c**) DNA on-target cleavage of *AAVS1-TS1* and *AAVS1-TS8* endogenous loci with CjCas9 WT and UltraCjCas9 after transfection. Data reported as mean ± standard deviation of indels percentages of n=3 biologically independent samples. Individual values are represented as empty circles.

Figure S7

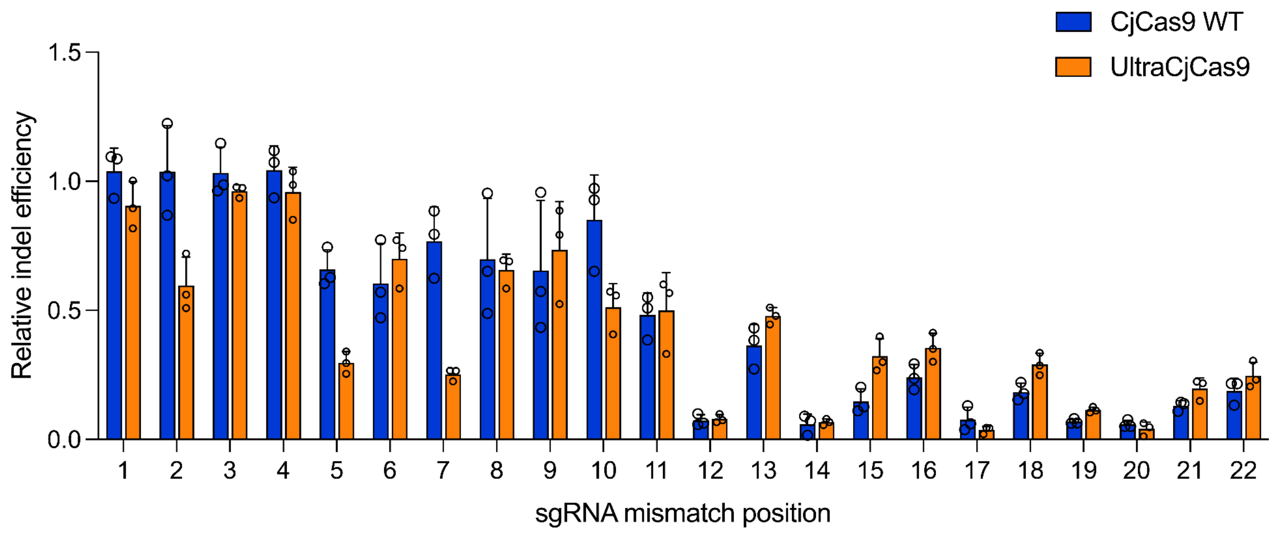

Figure S7. Editing activity of CjCas9 WT and UltraCjCas9 in the *PDCD1* locus using mismatched sgRNAs. Editing levels at the endogenous locus *PDCD1* obtained using CjCas9 WT and UltraCjCas9 with sgRNAs carrying single mismatches in each position from 1 to 22 relative to the PAM located in position 23-30. Relative indel efficiency is computed as indel frequencies obtained using mismatched guides normalized by the editing efficiency observed using the perfectly matched sgRNA. sgRNAs sequences are listed in **Additional file 2: Table S4**. Extended data reported in **Additional file 2: Table S11**. Data reported as mean ± standard deviation of relative indel efficiency of n=3 biologically independent samples. Individual values are represented as empty circles.
